# Impact of Doximity Residency Rankings on Emergency Medicine Applicant Rank Lists

**DOI:** 10.5811/westjem.2016.4.29750

**Published:** 2016-05-05

**Authors:** William J. Peterson, Laura R. Hopson, Sorabh Khandelwal, Melissa White, Fiona E. Gallahue, John Burkhardt, Aimee M. Rolston, Sally A. Santen

**Affiliations:** * University of Michigan, Department of Emergency Medicine, Ann Arbor, Michigan; † The Ohio State University College of Medicine, Department of Emergency Medicine, Columbus, Ohio; ‡ Emory University, Department of Emergency Medicine, Atlanta, Georgia; § University of Washington, Department of Emergency Medicine, Seattle, Washington; ¶ University of Michigan, Department of Obstetrics and Gynecology, Ann Arbor, Michigan

## Abstract

**Introduction:**

This study investigates the impact of the Doximity rankings on the rank list choices made by residency applicants in emergency medicine (EM).

**Methods:**

We sent an 11-item survey by email to all students who applied to EM residency programs at four different institutions representing diverse geographical regions. Students were asked questions about their perception of Doximity rankings and how it may have impacted their rank list decisions.

**Results:**

Response rate was 58% of 1,372 opened electronic surveys. This study found that a majority of medical students applying to residency in EM were aware of the Doximity rankings prior to submitting rank lists (67%). One-quarter of these applicants changed the number of programs and ranks of those programs when completing their rank list based on the Doximity rankings (26%). Though the absolute number of programs changed on the rank lists was small, the results demonstrate that the EM Doximity rankings impact applicant decision-making in ranking residency programs.

**Conclusion:**

While applicants do not find the Doximity rankings to be important compared to other factors in the application process, the Doximity rankings result in a small change in residency applicant ranking behavior. This unvalidated ranking, based principally on reputational data rather than objective outcome criteria, thus has the potential to be detrimental to students, programs, and the public. We feel it important for specialties to develop consensus around measurable training outcomes and provide freely accessible metrics for candidate education.

## INTRODUCTION

### Background

Influences on applicant rank lists have been well studied; however, the advent of the new Doximity ranking system may have introduced new considerations. Studies have shown that applicants base their decisions on a combination of personal factors including geographic location and quality of life, as well as program-specific factors including expected clinical experience, curriculum quality, interview day, experience with residents and faculty, and reputation of program.^1^–^5^ This process leads to an important decision that will impact the applicant’s future practice and location.^6^

In the 2014 application cycle, Doximity released residency program rankings by specialty in collaboration with *U.S. News and World Report*.^7^ Doximity is a free, HIPAA-compliant online platform for physicians’ social networking, collaboration and education. To create the residency rankings, Doximity administered a survey to their physician network in which they asked practicing physicians to “nominate up to 5 residency programs in your medical specialty that offer the best clinical training.^8^ ” More than 17,000 Doximity members responded to the survey, which resulted in a list of programs ranked by number based on majority vote. No independent consensus exists as to metrics for quality of training. The creation of a ranking system based on potentially biased responses from a selected group of physicians is controversial and has raised significant concerns, especially in the emergency medicine (EM) community.^9^

### Importance

The impact of *U.S. News and World Report* rankings on the undergraduate college application process has been well studied and has been shown to affect applicant decision-making, as well as public perception of universities and the funding that universities receive, regardless of debates about its accuracy. 10 Prior to the Doximity emergency medicine residency list being published, no central ranking system existed for residency applicants to refer to and to potentially impact their rank list.

### Goals of this Investigation

The effects of the Doximity findings, which have both reputational and ranking implications, are not well studied. This new ranking system may result in changes to applicants’ selections of residency programs. An initial study indicated that applicants are using Doximity in their choice of program applications. 11 This impact is potentially concerning, since there has been significant resistance to Doximity rankings in the EM community due to concerns about lack of objective criteria, inaccurate portrayal of residency programs, bias towards programs with larger alumni networks and provision of potentially misleading information to students as well as patients in the community. 12 The objective of this study is to investigate the impact of the Doximity rankings on the rank list choices made by residency applicants in EM.

## METHODS

### Study Design, Participants and Setting

Design was an 11-item survey emailed to all students who applied to EM residency programs at four different geographically diverse institutions: University of Michigan (Midwest), Ohio State University (Midwest), Emory University (South), and University of Washington (West). These email addresses were obtained through ERAS with the permission of ERAS.

### Methods, Measurements, and Outcomes

We assembled a research team consisting of two assistant deans, four residency program directors, a clerkship director, a resident, and a fourth-year medical student. For content validity, we modeled the questions after previous studies.^4^,^11^ The survey was reviewed by all authors with attention to response process and revised. For additional content validity, the questions were modeled after previous studies on a similar population (fourth-year students applying to residency). That survey was piloted by 20 residents and faculty and revised for response process validity. All authors reviewed this survey with attention to response process. The survey was emailed to students using a web-based platform, Qualtrics^™^, and student responses were anonymous. The survey was initially distributed at the beginning of March 2015 after rank lists were submitted by applicants and closed before Match Day. Three repeated requests were sent weekly to non-responders.

The survey asked first whether the student applicant was aware of or had looked at the Doximity rankings prior to submitting their rank list (Figure). Students who had looked at the rankings were eligible to complete the questions that assessed whether the Doximity rankings impacted their rank list construction. Students were also asked basic demographic information, how accurate they perceived the rankings to be on a 100-point scale (0 being not accurate at all and 100 being very accurate), and whether they increased or decreased the rank of programs based on the Doximity rankings. Additionally, space was provided for students to comment about the Doximity rankings. The comments were qualitatively reviewed and categorized into three groups: negative impression of rankings, neutral, and positive impressions of rankings. The negative category contained statements about how Doximity rankings were perceived as inaccurate or biased. The neutral category contained comments where students were unsure or did not care about the rankings. The positive category contained comments about how Doximity rankings were helpful or perceived as accurate. Finally, respondents were asked what factors affected their choice of programs.

### Analysis

Data analysis included descriptive statistics using SPSS 22. This study was determined to be exempt from institutional review board review at all four participating sites.

## RESULTS

### Characteristics of Study Subjects

We sent 1,641 emails to individual applicants for EM resident positions; 1,372 people opened the email, 850 started the survey, and 793 students completed the survey across the sample (overall response rate of 93% of people who started the survey, 58% of people who opened the email and 48% of total emails sent). The demographics of this sample of applicants who looked at the rankings were as follows: 63% male, 73% self-identified as White, 11% Asian, 5% Hispanic, 4% Black or African American, 1% American Indian, and 7% other. The regions of the institutions from which the applicants applied included 25% Northeast, 29% South, 27% Midwest, and 18% West. From the National Residency Matching Program for 2015, there were 1,613 U. S. senior applicants and 2,352 total applicants to EM.

### Main Results

Among the respondents, 531 students (67%) were aware of the Doximity rankings prior to submitting their rank lists. Among the students who were aware of the rankings, 359 (68%) looked at the rankings (Figure). Respondents found the Doximity rankings to be somewhat accurate with the mean score for accuracy of 41 (SD 23, range 0–100). Students were asked to “explain your assessment of the accuracy of the Doximity rankings.” Comments varied widely from “worthless” and “completely subjective” to “seems accurate” to “I don’t know.” Of the comments, 65% fell into the negative impressions category, 35% were neutral, and 10% were positive.

Of the students who looked at the rankings, 26% added programs to their rank list and 9.8% dropped programs from their rank list (Figure). The mean number of programs added per applicant who looked at the rankings was 1.2 (range 0 to >10) and the mean number of programs dropped was 0.3 (range 0 to 7). However, for those students who did add or drop programs to their rank list based on the rankings, the mean number added was 2.15 (SD 2.40) and dropped 1.31 (SD 1.07). Similarly, 26% of students increased the rank of programs on their rank list based on the Doximity rankings, and 19% decreased the rank of programs on their rank list. The mean number of programs that an applicant who looked at the Doximity rankings increased in rank was 0.6 programs (range 0 to 10), and the mean number of programs the applicant decreased in rank was 0.5 programs (range 0 to 8). For those students who changed their rank list based on the Doximity rankings, the mean increase in rank was 1.60 (SD 1.34) and decrease 1.46 (SD 1.14).

Students’ relative value of factors affecting residency preference are noted in Tables 1 and 2. They included preference for a particular geographic location, listed interview experience and experience with residents.

## DISCUSSION

This study found that a majority of medical students applying to residency in EM were aware of the Doximity rankings prior to submitting rank lists. A substantial number of applicants looked at the rankings and about a quarter of these applicants changed the number of programs and ranks of those programs when completing their rank list. Notably, the Doximity rankings were the least important factor compared to the other factors assessed in this study (Table 1). While these rankings were the least important, applicants did make changes in their rankings because of Doximity, demonstrating that the Doximity rankings may have some impact in applicant decision-making in ranking residency programs. A previous study similarly found that Doximity rankings affected the number of programs to which students applied. 13 We did not assess final match position of applicants, and without that information we cannot comment on how the Doximity rankings may have impacted final match position of applicants.

There has been significant resistance to the Doximity rankings in the EM community due to concerns about lack of objective criteria and inaccurate portrayal of residency programs. A consensus statement against the Doximity rankings endorsed by all major EM organizations was recently released in response to the rankings. The letter highlighted the significant threats to the validity of Doximity’s polling methods including the risk of sampling bias since EM physician survey responses were generated from Doximity members recruited through social media. 14 It further emphasized to applicants the importance of looking at programs for fit versus an arbitrary ranking system.

Despite the concerns expressed by the EM community and by students directly through their comments in our study, the existence of Doximity rankings allows students to make inferences about the reputation and value of programs based solely on these rankings and allows institutions to lay claim to reputation as well. It is well documented that reputation affects decision-making, and although the effect size is small, our study supports that applicant perception of reputation through rankings may impact their decision-making with residency rank lists.^15^,^16^ Medical students believe their program’s reputation will impact their future career prospects and the medical school faculty consider a school’s reputation in terms of their own visibility and the opportunities for career advancement, resources and research.

There is a strong interest on the part of students and EM programs for accurate, objective data about training programs. The inclusion of objective data could help guide applicants in selection of the best training environment for each learner. However, objective data for residency programs is limited and varies, and there is also the question of what data to include for a ranking system and more importantly, whether programs are willing to be transparent with certain information. Board passage rates were included in the Residency Navigator by Doximity for programs in internal medicine, family medicine, surgery and pediatrics. While markers like these are often considered by trainees and programs as indicators of successful training, these data speak to a single facet of training.

Our study also confirms the results of a previous study by Love and colleagues 4 that students are choosing programs based on personal factors such as geography and experiences. This may be due to the absence of objective data to assist decision-making. While it would be preferable to focus on objective data in lieu of rankings, we know from previous research (including this study) that rankings impact decision-making, and now Doximity has introduced an Internet-searchable residency ranking system that most applicants are aware exists. Perhaps efforts can be made to shift the way these rankings are generated and to promote searchable objective data about programs so that applicants can better identify the characteristics of programs that fit their individual interests and needs rather than creating an artificial roster of program superiority. Efforts to identify useful objective data, collect that data, and disseminate it in an easily navigable and Internet-searchable form could tremendously benefit student applicants by providing a set of metrics to evaluate and characterize programs in a transparent way. Other specialties have previously initiated work on this. 17 There is currently a study in progress looking to build consensus around a set of reportable metrics that may allow applicants to rank programs according to their needs and expectations of a program, such as percentage of grads who go on to academic practice.

## LIMITATIONS

Response rate is a limitation of this study, with only 793 students completing the survey out of an initial 1,641 students who were emailed, and may limit generalizability and provide response bias. It is possible that students chose to complete or not complete the survey based on preconceived perception of Doximity. Recall bias is another limitation in any survey-based study. Students may not remember exactly how Doximity affected their rank lists, as this survey was distributed after students had already submitted their lists. Another limitation is that students may not be able to fairly measure the impact of Doximity on their list choices since they do not have a personal comparison of applying prior to the release of Doximity.

## CONCLUSION

In conclusion, while applicants do not find the Doximity rankings to be important compared to other factors in the application process, the Doximity rankings result in a small change in residency applicant ranking behavior. This unvalidated ranking, based principally on reputational data rather than objective outcome criteria, thus has the potential to be detrimental to students, programs, and the public. We feel it important for specialties to develop consensus around measurable training outcomes and provide freely accessible metrics for candidate education. In addition, there should be a greater emphasis on student advising and matching to a best-fit program rather than to the most highly ranked one.

## Figures and Tables

**Figure:**
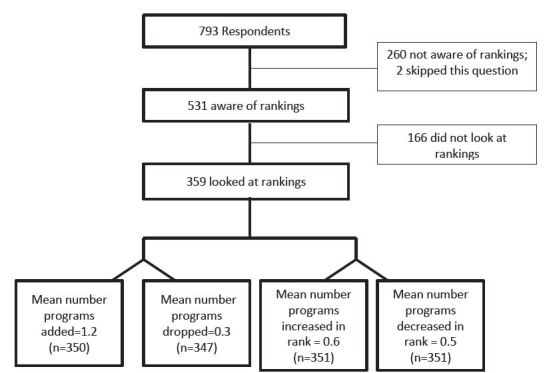
Applicants who looked at rankings.

**Table 1:** Factors of importance affecting choice of residency programs to which medical students applied.

	Number (%) n=772
Geographical preference	693 (90%)
Interview experience	636 (82%)
Experience with residents	596 (77%)
Proximity to spouse/significant other/family	439 (57%)
Doximity rankings	41 (5%)

**Table 2:** Factors important in making a rank list (rank those selected in question above).

Factor	Number of respondents placing factors in certain ranking				

	1^st^	2^nd^	3^rd^	4^th^	5^th^
	
Interview experience	183	174	134	30	0
Geographical preference	175	168	161	49	0
Doximity rankings	0	3	9	13	12
Experience with residents	109	158	141	78	4
Proximity to spouse/significant other/family	133	90	71	88	1
